# Tibialis Anterior muscle coherence during controlled voluntary activation in patients with spinal cord injury: diagnostic potential for muscle strength, gait and spasticity

**DOI:** 10.1186/1743-0003-11-23

**Published:** 2014-03-04

**Authors:** Elisabeth Bravo-Esteban, Julian Taylor, Manuel Aleixandre, Cristina Simon-Martínez, Diego Torricelli, José L Pons, Julio Gómez-Soriano

**Affiliations:** 1 Sensorimotor Function Group, Hospital Nacional de Parapléjicos, Toledo, Spain; 2 Spanish Natinal Research Council (CSIC), Madrid, Spain; 3 Nursing and Physiotherapy School, Castilla La mancha University, Toledo, Spain

**Keywords:** Electromyography, Intramuscular coherence, Spasticity, Isometric activation, WISCI II, AIS, Isokinetic activation, Residual voluntary motor function recovery, Neuroplasticity, Neurorehabilitation

## Abstract

**Background:**

Coherence estimation has been used as an indirect measure of voluntary neurocontrol of residual motor activity following spinal cord injury (SCI). Here intramuscular Tibialis Anterior (TA) coherence estimation was performed within specific frequency bands for the 10-60 Hz bandwidth during controlled ankle dorsiflexion in subjects with incomplete SCI with and without spasticity.

**Methods:**

In the first cohort study 15 non-injured and 14 motor incomplete SCI subjects were recruited to evaluate TA coherence during controlled movement. Specifically 15-30 Hz EMG was recorded during dorsiflexion with: i) isometric activation at 50, 75 and 100% of maximal voluntary torque (MVT), ii) isokinetic activation at 60 and 120°/s and iii) isotonic dorsiflexion at 50% MVT. Following identification of the motor tasks necessary for measurement of optimal TA coherence a second cohort was analyzed within the 10-16 Hz, 15-30 Hz, 24-40 Hz and 40-60 Hz bandwidths from 22 incomplete SCI subjects, with and without spasticity.

**Results:**

Intramuscular 40-60 Hz, but not 15-30 Hz TA, coherence calculated in SCI subjects during isometric activation at 100% of MVT was lower than the control group. In contrast only isometric activation at 100% of MVT 15-30 Hz TA coherence was higher in subjects with less severe SCI (AIS D vs. AIS C), and correlated functionally with dorsiflexion MVT. Higher TA coherence was observed for the SCI group during 120°/s isokinetic movement. In addition 15-30 Hz TA coherence calculated during isometric activation at 100% MVT or 120°/s isokinetic movement correlated moderately with walking function and time from SCI, respectively. Spasticity symptoms correlated negatively with coherence during isometric activation at 100% of MVT in all tested frequency bands, except for 15-30 Hz. Specifically, 10-16 Hz coherence correlated inversely with passive resistive torque to ankle dorsiflexion, while clinical measures of muscle hypertonia and spasm severity correlated inversely with 40-60 Hz.

**Conclusion:**

Analysis of intramuscular 15-30 Hz TA coherence during isometric activation at 100% of MVT is related to muscle strength and gait function following incomplete SCI. In contrast several spasticity symptoms correlated negatively with 10-16 Hz and 40-60 Hz TA coherence during isometric activation at 100% MVT. Validation of the diagnostic potential of TA coherence estimation as a reliable and comprehensive measure of muscle strength, gait and spasticity should facilitate SCI neurorehabilation.

## Introduction

Spinal cord injury (SCI) has a devastating impact on sensorimotor function, often leading to reduced quality of life, presenting a serious socioeconomic problem for national healthcare systems [1]. Although spinal damage is incomplete in approximately half of all SCI cases [2], only limited recovery of residual voluntary motor function is observed during the subacute phase of neurorehabilitation [3]. Indeed neurophysiological studies demonstrate only limited spontaneous recovery of voluntary motor function after incomplete SCI diagnosed with the American Spinal Injury Association Impairment Scale (AIS) [4]. Approximately 15-40% of individuals diagnosed with AIS B convert to AIS C, compared to 40% of subjects with AIS B SCI which convert to AIS D, and between 60-80% of AIS C which convert to AIS D [3]. Limited functional recovery could be mediated via several neuromotor control systems including automatic spinal motor control and descending corticospinal or extrapyramidal tract activity [5,6]. Furthermore the development of specific motor disorder symptoms associated with the spasticity syndrome [7,8] may further limit the recovery of voluntary motor strength, gait and activities of daily living [6-10]. As such the development of an objective and comprehensive measure of residual motor function recorded during subacute SCI neurorehabilitation which in turn reflects recovery or deterioration of descending or spinal neuromotor control mechanisms would help facilitate clinical diagnosis and improve treatment strategies. Ideally the neurophysiological measure should be clinically relevant, reflecting recovery of voluntary muscle strength and residual gait function during rehabilitation, while highlighting the development of debilitating central effects of problematic spasticity symptoms.

Electromyographic (EMG) muscle coherence estimation is a mathematical index that calculates the degree of synchronization of two independent EMG signal sources calculated in the frequency domain [11], and which can be obtained either within the same muscle (intramuscular coherence) [12,13], or between muscles [13-15]. Clinical studies have observed that measurement of synchronous motoneurone discharge (coherence) as an indirect measure of voluntary common drive is best recorded during isometric muscle contraction, and is significantly decreased following SCI [16]. Interestingly Hansen et al. [14] postulated that central common drive responsible for motor unit synchronization during walking may also be similar to that measured during tonic voluntary contraction. This analysis technique can also be applied to upper and lower limbs in subjects with central nervous system disorders, such as SCI or stroke, where the evidence also demonstrates reduced or absent motor unit synchrony during movement [16-18]. More recently the potential for muscle coherence analysis to demonstrate damage to voluntary motor control mechanisms and clinical function such as gait has been demonstrated in subjects with SCI [12,13].

Surprisingly no systematic studies are available that demonstrate the optimal testing conditions required to analyze motor unit synchronization during controlled movement, at moderate to strong muscle contractions or at slow and fast speeds, in subjects with residual voluntary muscle strength and gait function after incomplete SCI. Several physiological studies justify the application of muscle coherence estimation as an indirect measure of voluntary motor drive with respect to specific motor tasks. A relationship between the force of isometric contraction [16] and corticospinal activation has been alluded to in man [19], based partly on the observation of reduced intracortical inhibition [20] which in turn can be modulated by muscle strength training [21]. With respect to the velocity of movement, a study in subjects with SCI also alludes to a relationship between gait velocity and corticospinal tract activity which was calculated as intramuscular 10–20 Hz TA coherence activity, although the relationship between these measures were related to their common correlation with foot drop [13].

Spinal cord injury invariably leads to different degrees of corticospinal tract injury [3]. Given that EMG coherence activity is related to corticospinal tract function [11,13] this technique has been used in several research studies to indirectly address the state of descending motor control mechanisms. As an example, Hansen [22] observed an alteration in muscle coherence related to walking dysfunction. Several clinical neurophysiological studies have also proposed that muscle coherence activity calculated within specific frequency bands reflects activity of different neuronal systems, where high frequency activity (15–30, 24-40 Hz) [23] may reflect descending neuromotor control [15], compared to low frequency coherence (1-12 Hz) which may be associated with spinal activity [24-26]. Importantly for the diagnosis of SCI, coherence activity within the 15-30 Hz range has been estimated as a standard indirect measure of pyramidal tract integrity [27-29].

Measurement of residual voluntary activity within the Tibialis Anterior (TA) muscle may represent an interesting diagnostic marker of function after SCI, particularly as this muscle receives strong innervation from the corticospinal system [30]. Indeed ankle dorsiflexion has been used to indirectly measure central adaptative neuroplasticity of the corticospinal tract during rehabilitation [31,32], while the detection of TA coactivation during plantarflexion can also detect the development of maladaptive mechanisms after incomplete SCI, such as specific symptoms of spasticity [6]. Although spasticity has been reported in up to 78% of chronic SCI individuals [10,33-35] it is not clear whether the presence of symptoms such as muscle hypertonia or spasms directly affect the central neuronal drive that mediates the limited recovery of residual motor function [6,36]. Therefore analysis of intramuscular TA coherence could represent a comprehensive measure of functional change after SCI during subacute rehabilitation.

Systematic evaluation of the optimal movement testing conditions for muscle coherence analysis and the intramuscular TA frequency band best related to residual voluntary muscle strength and gait function should provide important orientative data to guide the diagnostic potential of monitoring motor unit synchronization as a comprehensive diagnostic tool for clinical motor function after incomplete SCI.

## Methods

The study was divided into two trials. The objective of the first trial was to identify the optimal specific kinematic condition to measure differences in motor unit synchronization between healthy non-injured subjects and individuals with incomplete SCI (Table 1). Therefore standard intramuscular TA coherence estimation within the 15-30 Hz band [27-29] during different types of controlled dorsiflexion movement was performed. Once the optimal movement tasks were identified a second trial was performed on a larger cohort of patients (Table 2) with an additional emphasis on analyzing TA coherence within different frequency ranges within the total 10-60 Hz bandwidth. The selection of these frequency bands was based on the standard 15-30 Hz frequency band [27-29] and studies that have implicated a physiological significance to low frequency [24-26] and high frequency coherence [37]. In addition, the second trial investigated the impact of specific spasticity symptoms on TA coherence calculated during the optimal controlled test movement protocols.

**Table 1:** Individual SCI characteristics for Cohort I

	**Age (Years)**	**Gender**	**Etiology**	**Level**	**Time (Weeks)**	**MT**	**DMVT (Nm)**
**1**	25	M	T	C4	22	4+	105
**2**	64	M	T	C4	480	4	112
**3**	27	M	T	C5	4	4	82
**4**	43	F	T	C6	40	4	70
**5**	59	F	NT	T3	28	3	73
**6**	45	F	NT	T7	20	4	110
**7**	21	M	NT	T7	14	4	103
**8**	22	M	T	T7	14	4	55
**9**	44	M	T	L1	36	3	115
**10**	41	M	T	C6	16	5	150
**11**	31	M	NT	C6	4	3	208
**12**	30	M	T	C6	12	4+	179
**13**	26	M	T	D7	6	4	84
**14**	30	M	T	D12	6	3	65

M: male; F: female; T: traumatic; NT: non-traumatic; Level: neurological level of injury; Time: time from injury (weeks); MT: Tibialis Anterior muscle testing score; DMVT: dorsiflexion maximal voluntary torque (Nm).

**Table 2:** Individual SCI characteristics for Cohort II

	**Gender**	**Age (Years)**	**Asia**	**Level**	**Etiology**	**Ashw**	**Penn**	**Time (Weeks)**
**1**	F	40	C	C4	NT	0	0	10
**2**	M	55	C	C5	NT	0	0	10
**3**	F	68	C	T8	NT	0	0	20
**4**	F	70	C	T10	NT	0	0	10
**5**	F	64	C	T12	NT	0	0	22
**6**	M	36	D	C3	NT	1	0	8
**7**	M	40	D	T11	T	0	0	18
**8**	M	57	C	C4	T	3	1	24
**9**	M	62	C	C5	T	7	2	48
**10**	M	26	C	C5	T	3	1	20
**11**	M	57	C	T6	T	9	3	20
**12**	M	22	C	T7	T	1	1	24
**13**	M	54	C	T7	T	3	1	28
**14**	F	68	C	T8	NT	1	2	12
**15**	M	70	C	T11	T	5	1	24
**16**	M	37	D	C2	T	6	1	18
**17**	M	62	D	C4	T	2	1	40
**18**	M	25	D	C4	T	2	1	48
**19**	M	48	D	C4	NT	2	1	8
**20**	M	36	D	C5	T	4	3	4
**21**	M	63	D	C5	T	4	2	16
**22**	M	45	D	C7	T	2	1	8

M: male; F: female; T: traumatic; NT: non-traumatic; Level: neurological level of injury; ASHW: total modified Ashworth score calculated for the knee and ankle joints during flexion and extension movement; Penn: Penn spasm scale; Time: time from injury (weeks).

### Subjects

The study was performed following approval by the Local Toledo Hospital Clinical Ethical Committee (number of approval 152, 2012). All recruited subjects signed a consent form and gave their permission for data publication. Healthy non-injured subjects (n=15) and individuals with incomplete motor SCI (n=14) with a TA and Triceps Surae muscle score ≥2 [38], were recruited in the first trial (SCI cohort I). In the second trial (cohort II) 22 subjects were recruited with the same inclusion criteria. Patients were diagnosed with spasticity if they presented a modified Ashworth score [39] >1 and/or Penn score [40] ≥ 1. The exclusion criteria included diagnosis of musculoskeletal or peripheral nervous system disorders.

### Controlled movement tasks

Analysis of TA coherence was carried out during controlled movement with the subjects comfortably seated in a dynamometer (KinCom, Chattanooga Group Inc.). The trunk and pelvis of the tested leg were supported using straps. The hip, knee and the ankle joints were flexed at 90°. In SCI subjects the criteria included muscle activity calculated from the global muscle score TA muscle score greater than 2. Consequently the leg was capable of generating moderate contraction levels to perform the controlled movement protocols which also avoided methodological problems associated with coherence analysis with non-rectified signals [41]. For the non-injured control subjects the TA of the right leg was recorded for coherence estimation. The EMG activity was recorded using double differential surface electrodes, at a preamplifier gain of 10 V/V and open bandwidth (Delsys Inc. Signal Conditioning Electrodes 3.1), placed in two specific locations on the TA muscle belly, and separated by a minimum of 10 cm to avoid electrical cross-talk [12,14,42]. In the first trial 15 healthy subjects and 14 individuals with SCI (cohort I) performed three different types of controlled TA muscle movement tasks: i) two isometric activations maintained for 5 s, at 50%, 75% and 100% of the maximal voluntary torque (MVT) level, recorded with the dynamometer; ii) five cycles of isotonic activation with a range of motion from 30° plantarflexion to 0° dorsiflexion, with the required active force set to 50% of MVT; iii) ten cycles of isokinetic activation at 60°/s and 120°/s, from 30° plantarflexion to 0° dorsiflexion. All the controlled movement tasks, with the exception of the 100% MVT isometric activation, were randomized. The comprehensive movement testing conditions precluded the analysis of long EMG segments (see below).

In the second trial 22 SCI subjects (15 of whom presented spasticity) performed two types of controlled TA activation i) two isometric activation at 100% of MVT maintenance for 5 s and ii) ten cycles of isokinetic movement at 60°/s and 120°/s, from 30° plantarflexion to 0° dorsiflexion. In addition several clinical tests were applied in the second trial including i) gait function measured with the WISCI II [43], ii) the modified Ashworth scale to measure muscle hypertonia in the knee and ankle joint during flexion-extension movement, iii) spasm frequency quantified with the Penn scale and iv) severity of evoked spasms measured with the spinal cord assessment tool for spastic reflexes scale (SCATS) [44]. Passive resistive torque to ankle dorsiflexion was also tested in the second cohort at slow (30°/s) and fast (120°/s) movements in order to evaluate both the viscoelastic and reflexive components respectively of muscle hypertonia [45]. Passive torque measures were obtained with the subject seated in the dynamometer, with the hip joint flexed at 90° and the knee joint at 10°, and obtained from ten ankle joint mobilizations from 30° plantarflexion to 0° dorsiflexion at 30°/s and 120°/s. EMG data was collected from 21/22 subjects during isometric activation at 100% of MVT, with data not recorded from 1/15 of the 15 spastic subjects. Correlation between intramuscular TA coherence during isometric activation at 100% of MVT and spasticity measures was performed for the 14 patients with hypertonia or spasm activity [6,10].

### Data analysis and statistical evaluation

Coherence is a measure of how closely the two EMG signals are related by a linear transformation [46]. Coherence is estimated between 0–1, where a value of 1 indicates that the two signals are highly correlated, while a value of 0 means that both signals are independent. Electromyographic signals were recorded with a 10 KHz sampling frequency (MicroPlus 1401, Cambridge Electronic Design) and were subsequently down sampled to 2 KHz using a low pass filter of 700 Hz to avoid aliasing (Matlab 7.11). Muscle coherence activity was calculated with the Signal Processing Toolbox of Matlab 7.11 by estimating the power spectral densities with Welch’s method [47]. Due to the methodological requirements of measuring intramuscular TA coherence during several controlled movement tasks in subjects with SCI, the criteria of recording EMG signals of at least 3.5 seconds was observed for all subjects and movement tasks. The signal was divided into 8 data segments with 50% overlapping segments performed with a Hamming window [48]. To obtain the coherence in each frequency band (10–16, 15–30, 24–40, and 40–60 Hz), all the coherences points within each specific band from each subject were averaged to obtain the grand average coherence for the specific frequency band.

There is some controversy regarding the requirement of rectifying EMG signal activity for coherence analysis with studies for [49-51] and against this process [52,53]. Indeed rectification of EMG activity amplifies the power spectrum of lower frequencies [49,54] and more clearly provides information regarding neuronal firing timings to assess the general activity envelope [53]. However rectification of EMG activity for coherence analysis has been demonstrated to be necessary at low muscle contraction levels [41]. This suggests coherence estimation of the moderate to strong muscle activity levels recorded in this study may not be affected by collection of non-rectified EMG signals and may not present problems in identifying common inputs to motoneurones in general [41]. Nevertheless rectification is preferable to reject other artefacts during coherence analysis [55].

The velocity dependence of intramuscular TA coherence was calculated by calculating the ratio of its value during isokinetic TA muscle movement at 120°/s and at 60°/s. Statistical analysis was performed with a commercial software package (SigmaStat version 3.1, Systat software, Inc, USA). Due to the non normal distribution of the data, non-parametric tests were adopted. The Kruskall Wallis test was used to compare different controlled muscle activation protocols within the first SCI cohort. The Mann–Whitney test was used to compare intramuscular TA coherence between the healthy non-injured group with both SCI cohorts (Median and 25th-75th Percentiles) and to analyze differences between SCI subjects with and without spasticity in the second cohort. The Spearman correlation test was used to identify the relationship between TA coherence with MVT, gait function and spasticity measures. Statistical significance was defined as p ≤ 0.05, with trends described at p ≤ 0.07.

## Results

### Clinical characteristics of subjects

Fifteen healthy subjects (8 male) with a median age of 26.6 years (23.2-28.3, 25th percentile–75th percentile range) and 14 subjects with SCI (11 male) with a median age of 30.5 (26.4-44.3) years (SCI cohort I, Table 1), were recruited for the study of intramuscular TA coherence during different controlled muscle activation protocols. A difference in age was found between the healthy subjects and the individuals with SCI in cohort I, (p = 0.032) but no differences in gender were found. In the second larger SCI cohort an additional 22 subjects with SCI (17 male) with a median age of 54.5 (37.0–63.0) were enrolled for the more specific analysis of the relationship between frequency-specific TA coherence estimation and residual voluntary muscle strength, gait and spasticity (Table 2). This patient group were older than SCI subjects from cohort I (p = 0.018) and also showed significant differences with respect to the non-injured group p = 0.001). Fifteen of the subjects in the second cohort were diagnosed with spasticity. Importantly no significant difference between the SCI clinical characteristics were identified between cohorts I and II for gender, time from injury or maximal dorsiflexion torque (Tables 1 and 2).

### 15-30 Hz TA coherence activity during controlled movement in subjects with or without SCI

**Figure 1:**
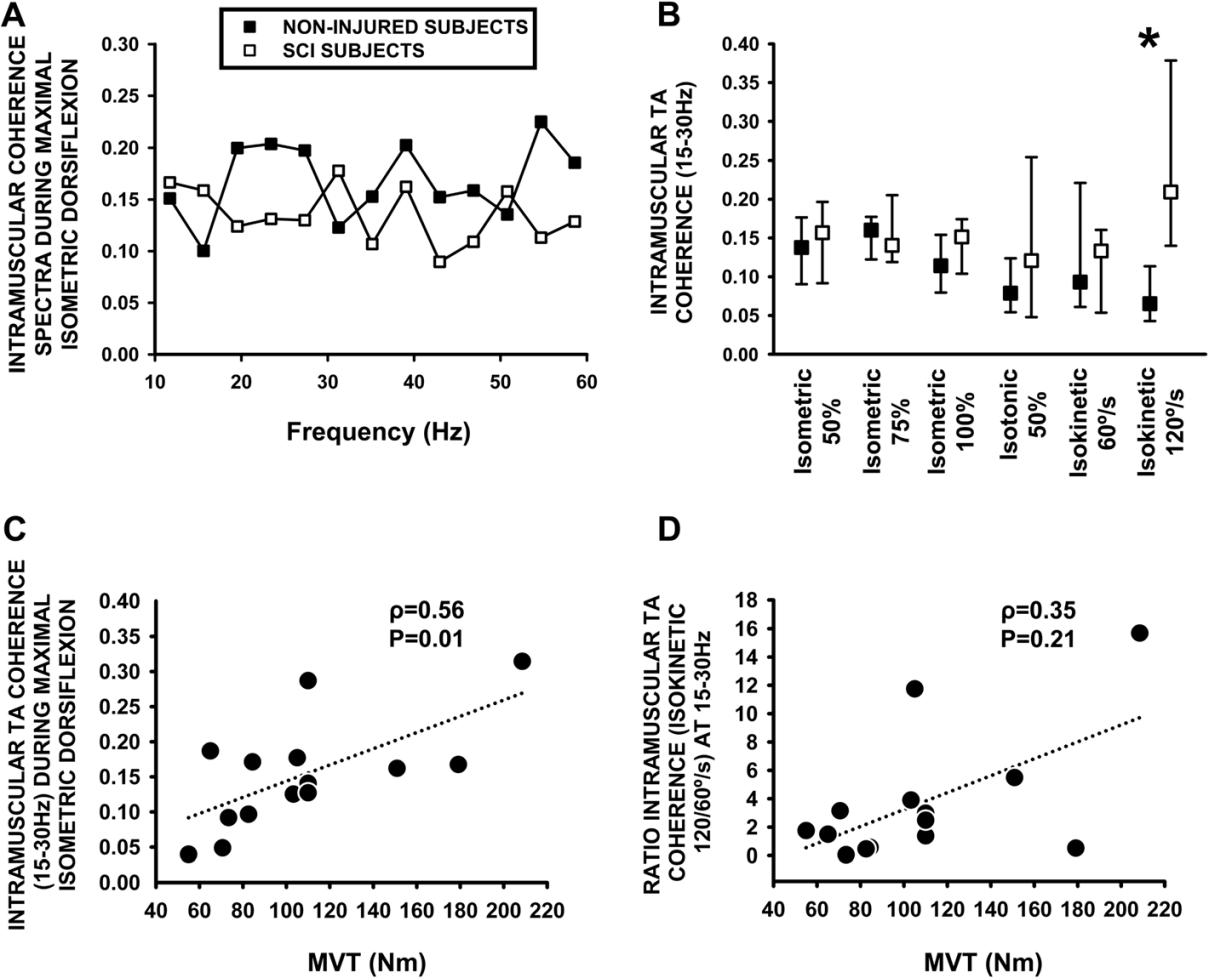
**Intramuscular TA coherence in the SCI group and in the non-injured group. A**: Intramuscular TA coherence spectra (10-60 Hz) calculated during maximal isometric dorsiflexion in the SCI group (white symbols) and in the non-injured group (black symbols). **B**: Analysis of 15-30 Hz intramuscular TA coherence from healthy subjects and individuals with SCI during isometric, isotonic and isokinetic muscle activation. *: p ≤ 0.05. **C**: Correlation between intramuscular TA coherence calculated during isometric activation at 100% of MVT with maximal voluntary dorsiflexion torque. **D**: Correlation between velocity-dependent intramuscular TA coherence during 120/60°/s isokinetic activation with maximal voluntary dorsiflexion torque.

**Figure 2:**
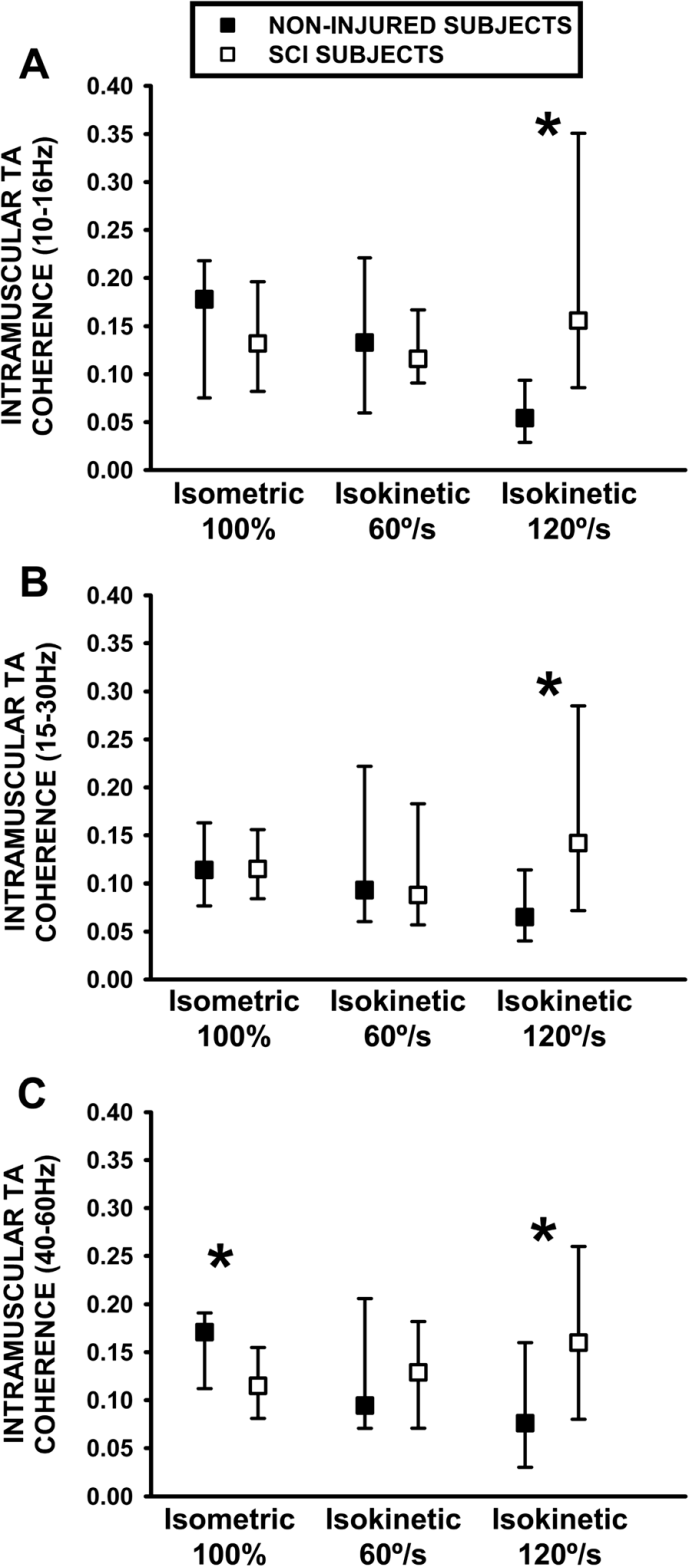
**Analysis of 10-16 Hz, 15-30 Hz and 40-60 Hz TA coherence from healthy subjects and individuals with SCI during isometric, isotonic and isokinetic muscle activation. A:** 10-16 Hz intramuscular TA coherence. **B:** 15-30 Hz intramuscular TA coherence. **C:** 40-60 Hz intramuscular TA coherence. *: p ≤ 0.05.

Analysis of the complete coherence spectra for 10-60 Hz recoreded during maximal isometric activation in subjects with SCI and non-injured control subjects (Figure 1A) suggested that activity within the 15–30 and 40–60 Hz bands was higher for the non-injured subjects compared to the SCI individuals, and minor differences within the 10–16 Hz band suggesting greater activity in subjects with SCI. Intramuscular TA muscle coherence calculated within the 15–30 Hz frequency band and compared between non-injured healthy subjects and individuals with SCI (cohort I, Table 1) revealed no significant differences during isotonic or isometric dorsiflexion at 50%, 75% or 100% of the MVT (Figure 1B). However 15-30 Hz TA coherence activity calculated during isometric activation at 100% of MVT in the first SCI cohort correlated positively with MVT during dorsiflexion (ρ = 0.56, p = 0.01, Figure 1C), although this correlation was not present in the non-injured healthy group (ρ = 0.32, p = 0.23). The level of 15-30 Hz TA coherence activity calculated during isokinetic contraction at 60°/s showed no differences between non-injured and SCI group. Nevertheless TA coherence activity during isokinetic activation at 120°/s revealed higher values during fast muscle activation in the SCI cohort I group (0.20, 0.13-0.38) when compared to the non-injured group (0.06, 0.04-0.11, p = <0.001; Figure 1B). Calculation of the ratio of TA coherence for isokinetic activation at 120/60°/s revealed higher values for the 15-30 Hz band for the first SCI cohort I (2.1, 0.6-3.9) when compared to the non-injured healthy group (0.58, 0.35-1.00, p = 0.029). No relationship was identified between the velocity-dependent 15-30 Hz TA coherence ratio and dorsiflexion MVT in the first SCI cohort (Figure 1D). Following identification of differences of intramuscular TA coherence activity during isometric activation at 100% of MVT and at faster isokinetic movement, an analysis of the physiological significance of each of the selected coherence frequency bands was sought with the optimal kinetic tasks in the second cohort.Although no differences were identified for the 10-16 Hz (Figure 2A) or 15-30 Hz TA coherence bands (Figure 2B) between healthy and SCI groups during isometric activation at 100% of MVT in the second SCI cohort, calculation of the 40-60 Hz band revealed a lower level of activity in SCI subjects (0.11, 0.08-0.16) compared to the non-injured group (0.17, 0.11-0.19; p = 0.05. Figure 2C). The velocity-dependence of TA coherence activity in subjects with SCI was corroborated in the second cohort for most of the frequency bands between 10-60 Hz (Figure 2A-C). In the 15-30 Hz bandwidth, the difference for intramuscular TA coherence compared to the non-injured group during isokinetic activation at 120°/s from the first cohort were replicated in the second cohort with SCI subjects (0.14, 0.07-0.28) when compared to healthy subjects (0.065, 0.04-0.11; p = 0.01, Figure 2B) and for differences between the 120°/s/60°/s ratio calculation in the SCI group (1.5, 0.8-2.02) compared to the non-injured group (0.59, 0.37-1.87; p = 0.04). Moreover the 10-16 Hz activity revealed higher TA coherence during isokinetic activation at 120°/s (Figure 2A) in SCI subjects (0.16, 0.09-0.35) with respect to non-injured subjects (0.08, 0.03-0.16; p = 0.05). Higher 40-60 Hz TA coherence was also identified in SCI subjects during isokinetic activation at 120°/s (0.16, 0.08-0.26, Figure 2C) and with the 120/60°/s ratio (1.64, 0.92-2.5) compared to the non-injured subjects (0.08, 0.03-0.16; p = 0.05 and 0.8, 0.31-1.8; p = 0.06 respectively).

### TA muscle coherence, muscle strength and gait function after spinal cord injury

The functional relationship between TA coherence calculated during isometric or isokinetic activation was investigated by assessing muscle strength and gait function in the second larger SCI cohort, with a special emphasis on analysis of the frequency specific bandwidths (n = 22, Table 2). As such the positive association observed between TA coherence during isometric activation at 100% of MVT and dorsiflexion MVT was corroborated for both the 15–30 Hz (replicating results from the first SCI cohort) and 24-40 Hz frequency bands (Table 3). Moreover 15-30 Hz TA coherence estimated during isometric activation at 100% of MVT correlated with the grade of residual gait function in subjects with SCI (ρ = 0.41, p = 0.05), with a trend present for coherence activity within the 24-40 Hz bandwidth (Table 3). TA muscle coherence calculated as the 120/60°/s ratio failed to correlate with residual MVT or gait function in the second cohort (data not shown).

**Table 3:** **Correlation between isometric activation at 100**% **of MVT intramuscular TA coherence with residual muscle strength and gait function in subjects with SCI in Cohort II**

**Frequency ranges (Hz)**	**MVT (Nm)**	**WISCI II (21 points)**
10-16 Hz	0.07 p = 0.74	0.26 p = 0.24
15-30 Hz	**0.50 p = 0.01**	**0.41 p = 0.05**
24-40 Hz	**0.41 p = 0.05**	*0.40 p = 0.06*
40-60 Hz	0.01 p = 0.95	0.36 p = 0.10

MVT (Nm): maximal voluntary torque; TA: Tibialis Anterior. n = 21. p ≤ 0.05 in bold and p ≤ 0.06 in italics.

### TA muscle coherence, time of evolution and severity of incomplete SCI

**Figure 3:**
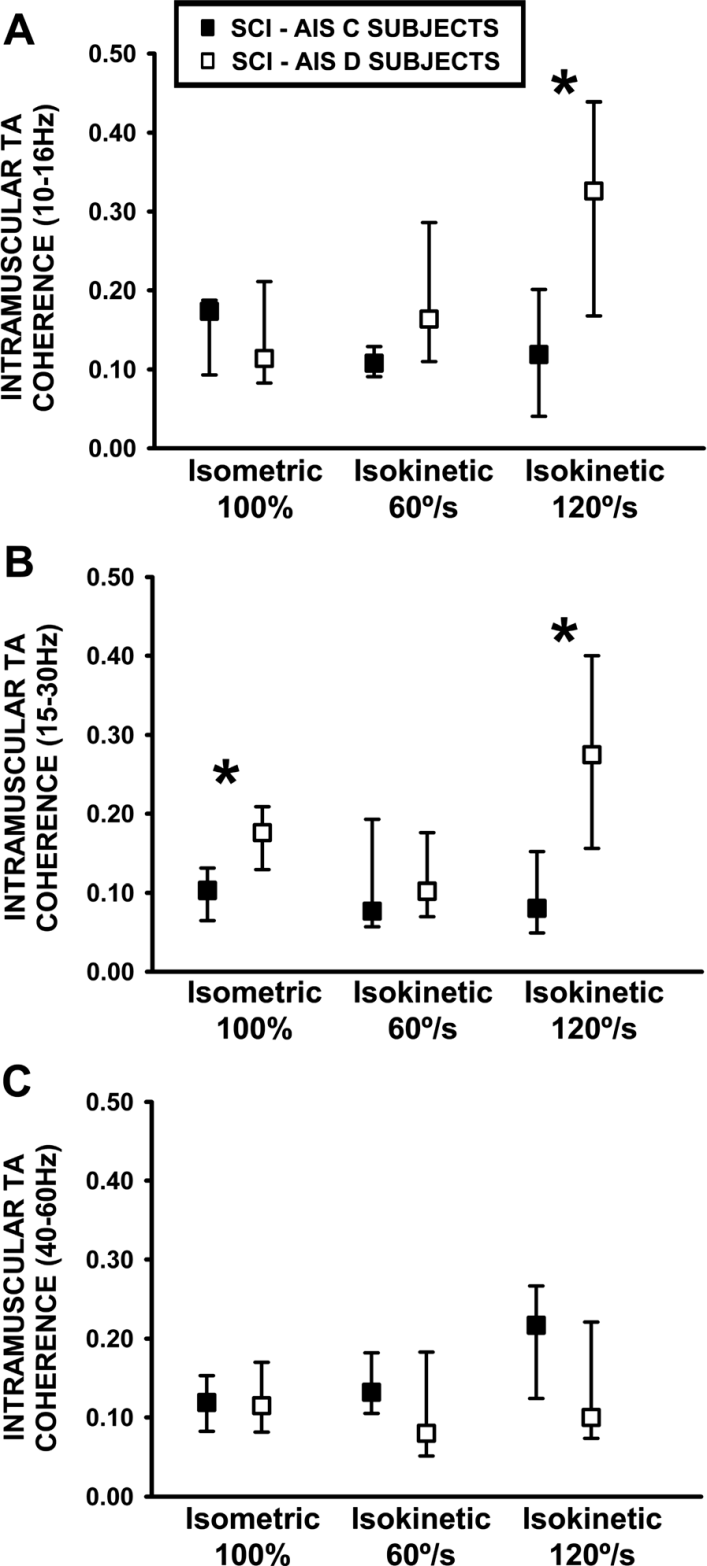
**Analysis of 10-16 Hz, 15-30 Hz and 40-60 Hz TA coherence activity from SCI subjects with AIS C and AIS D during different types of muscle activation. A**: 10-16 Hz intramuscular TA coherence. **B**: 15-30 Hz intramuscular TA coherence. **C**: 40-60 Hz intramuscular TA coherence. *: p ≤ 0.05.

Intramuscular 15-30 Hz TA coherence calculated either during maximal isometric or isokinetic activation in subjects with SCI recruited from the second cohort (Table 2) also revealed differences in individuals diagnosed according to AIS severity and time from injury. In subjects diagnosed as AIS D, higher 15–30 Hz TA coherence was estimated during isometric activation at 100% of MVT (0.17, 0.12-0.20) when compared to individuals diagnosed with a SCI grade of AIS C (0.13, 0.06-0.13; p = 0.019. Figure 3B). Furthermore 15-30 Hz TA coherence activity estimated during 120°/s isokinetic activation was higher in subjects with AIS D (0.27, 0.15-0.40) compared to AIS C (0.08, 0.40-0.15, p = 0.015) (Figure 3B) and also for the 10-16 Hz frequency band (AIS D, 0.33, 0.04-0.20 compared to AIS C 0.12, 0.17-0.44; p = 0.035 Figure 3A). This was also the case when the ratio of 15-30 Hz TA coherence was calculated during isokinetic activation for the 120/60°/s ratio; AIS D (1.9, 1.7-3.4) and AIS C (1.00, 0.6-1.4, p = 0.019). No differences were found for the other movement tasks or specific coherence frequency bands. Finally a positive correlation was determined between the TA coherence calculated as the 120/60°/s ratio within the 24-40 Hz frequency band and the time from SCI, significantly for the first cohort (ρ = 0.54, p = 0.05) and as a trend for the second SCI cohort (ρ = 0.50, p = 0.07).

### TA coherence and specific spasticity symptoms after spinal cord injury

**Figure 4:**
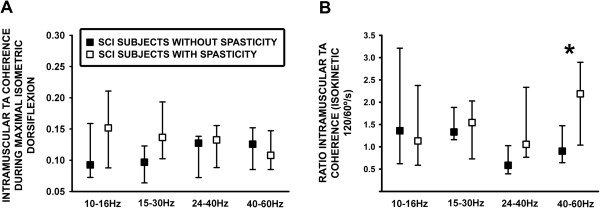
**Analysis of specific TA coherence activity within the total 10-60 Hz bandwidth from subjects with and without clinically diagnosed spasticity. A**: Intramuscular TA coherence estimated during isometric activation at 100% of MVT. **B**: Ratio of intramuscular TA coherence estimated during isokinetic movement at 120/60°/s. *p ≤ 0.05.

Analysis of the impact of spasticity on intramuscular TA coherence was performed in 14 subjects with SCI in the second cohort compared to seven subjects without SCI spasticity (Table 4 and Figure 4A and 4B). In general no differences were identified for TA coherence observed during isometric activation at 100% of MVT for subjects diagnosed without or with spasticity within the 10–16 Hz, 15–30 Hz, 24–40 Hz or 40–60 Hz bands (Figure 3A) eventhough a non-significant higher level of coherence activity was identified within the lower frequency band in the SCI spasticity group. TA coherence calculated as the ratio of 120/60°/s isokinetic activation was higher in the group with SCI spasticity (2.18, 1.03-2.89, p = 0.05) compared to individuals without spasticity (0.90, 0.64-1.47, Figure 4B).

**Table 4:** Correlational analysis between intramuscular TA coherence calculated during isometric activation at 100% of MVT with spasticity measures in subjects with SCI in Cohort II

**Frequency ranges**	**Ashw**	**PRT 30°/s**	**PRT 120°/s**	**Penn**	**SCATS**	**Clonus**
10-16 Hz	0.22 p = 0.45	*−***0.58 p = 0.04**	**−0.59 p = 0.03**	0.03 p = 0.90	−0.05 p = 0.80	**0.58 p = 0.04**
15-30 Hz	*−0.30 p = 0.09*	−0.41 p = 0.17	−0.39 p = 0.19	*−0.53 p = 0.06*	−0.18 p = 0.54	0.25 p = 0.41
24-40 Hz	**−0.58 p = 0.02**	−0.15 p = 0.61	−0.17 p = 0.57	−0.18 p = 0.55	−0.33 p = 0.27	−0.41 p = 0.16
40-60 Hz	**−0.60 p = 0.02**	−0.26 p = 0.38	−0.23 p = 0.45	0.16 p = 0.60	**−0.56 p = 0.05**	−0.04 p = 0.88

MVT (Nm): maximal voluntary torque. Ashw: Ashworth scale during knee flexion. PRT: passive resistive torque to dorsiflexion. SCATS: total flexor and extensor spasm. n = 21. p ≤ 0.05 in bold and p ≤ 0.06 in italics.

Correlation analysis of the relationship between TA coherence activity estimated during isometric activation at 100% of MVT with specific symptoms of SCI spasticity generally revealed a negative effect of muscle hypertonia, passive resistive torque, and involuntary muscle contractions within different bandwidths (Table 4). Specifically, passive resistive torque to ankle dorsiflexion in subjects with spasticity measured at 30°/s (ρ = −0.58, p = 0.04) or 120°/s (ρ = −0.59, p = 0.03) revealed a negative correlation with 10-16 Hz TA coherence. In contrast, the modified Ashworth scores correlated negatively with TA coherence within the higher 24-60 Hz TA frequency band (Table 4). The severity of evoked spasms measured with the SCATS also highlighted a negative relationship for high frequency 40-60 Hz TA coherence (ρ = −0.56, p = 0.05) and an inverse correlation trend was also present for the Penn score but only within the 15-30 Hz bandwidth. In contrast, a positive correlation was identified between the degree of clonus activity and TA coherence estimated during isometric activation at 100% of MVT within the low frequency 10–16 Hz bandwidth.

## Discussion

This is the first study that has systematically analyzed intramuscular TA coherence from subjects with incomplete SCI during different movement tasks and within specific frequency bands between 10-60 Hz. Comprehensive analysis highlighted the functional and clinical relationship between specific TA muscle coherence activity with residual voluntary dorsiflexion strength, clinical characteristics of SCI including the severity and time from injury, and the negative impact of different spasticity signs. Estimation of standard 15–30 Hz TA coherence during maximal isometric contraction or fast (120°/s) isokinetic movement was related to several functional and clinical parameters in subjects with SCI, while a novel change in 40–60 Hz coherence was identified specifically in subjects with clinical measures of SCI spasticity. The identification of the optimal task and frequency bands required to support the physiological and functional significance of TA coherence activity should facilitate the diagnosis of motor recovery mediated by central motor control mechanisms, in addition to detecting early signs of adaptive and maladaptive neuroplasticity during subacute neurorehabilitation after incomplete SCI.

### TA coherence estimation: Relationship with strength and velocity of voluntary muscle contraction after SCI

Several clinical studies have demonstrated that 15-30 Hz muscle coherence reflect neural activation of muscle function via cortical activation of synchronous motor units in antagonist muscles [28,56]. Indeed both intramuscular and intermuscular coherence is often regarded as an indirect measure of corticospinal activity [56-58]. In this study isometric activation at 100% of MVT was identified as the best controlled movement task to demonstrate higher 15-30 Hz TA motor unit synchronisation in subjects clinically graded with less severe SCI, but not when compared to the non-injured group (c.f. 40-60 Hz activity). Muscle coherence estimation has previously been estimated in the lower limb of healthy subjects, especially during tasks that involve co-contraction of lower limb muscles at the same joint, such as with balance [17], a motor task mediated by a subpopulation of the total corticospinal system [6]. In other studies isometric contraction has demonstrated changes in motor unit synchronisation following SCI or motoneuron disease [15,27,29].

In the present study several consistent correlations indicated that 15-30 Hz TA coherence calculated during isometric activation at 100% of MVT correlated significantly with muscle strength following SCI. The reduction or absence of lower limb muscle coherence with an associated loss of lower limb muscle strength following damage to the corticospinal pathway has been identified at these frequencies [11,22,25,26]. Furthermore recovery of corticospinal tract function in subjects with incomplete SCI during intensive locomotor training has been measured directly neurophysiologically in parallel with an increase in TA motor unit synchronisation [15], suggesting that estimations of muscle coherence may approximate descending motor control function.

Systematic analysis of TA coherence in this study also revealed consistently higher values for all the frequency bands when calculated during fast isokinetic dorsiflexion in subjects with SCI, compared to healthy subjects. However when TA motor unit synchronisation was compared within subjects with different grades of SCI, higher velocity-dependent coherence activity was observed in subjects with a more incomplete SCI, but only within the 10-16 Hz and 15-30 Hz bandwidths. In this regard, recovery of cortical motor evoked potential amplitude during subacute SCI correlated only with maximal movement velocity of dorsiflexion rather than maximal isometric muscle strength in general [31,32]. Indeed hyperexcitability of the corticospinal system demonstrated during task dependent movement conditions may reflect cortical compensation for the functional deficit produced following SCI [59] leading to higher coherence activity in subjects with mild AIS scores.

This study also supports the relatively new identification of the physiological relevance of 40-60 Hz EMG coherence activity [37]. Lower 40-60 Hz TA coherence activity observed in subjects with SCI during isometric activation at 100% of MVT in the second cohort was clear, but no functional correlation was identified with this high frequency TA coherence activity and muscle strength, gait or SCI severity. Coherence activity within the 40-60 Hz band has been associated with non-pyramidal tract neuronal activity possibly related to residual activity within either reticulospinal and/or propriospinal tracts following SCI [37] or following motor neurone disease [27]. Both these studies suggest that measurement of high-frequency activity may lead to important physiological information regarding mechanisms of functional recovery unrelated to corticospinal neuroplasticity after SCI [27,37].

### TA coherence estimation as an approximation of residual clinical motor function after SCI

Residual voluntary motor function after SCI is initially diagnosed with the AIS scale, which characterises AIS C from AIS D with the presence of useful motor activity but without normal strength or gait [4]. Furthermore both SCI grades are associated with a different degree of motor recovery throughout the course of subacute SCI [3], which is usually corroborated with neurophysiological testing of corticospinal motor evoked potentials [60]. The results from our study also suggest that the estimation of 15-30 Hz TA coherence during isometric contraction may be useful as an approximation of corticospinal function and SCI grade based on higher motor unit synchronization in subjects diagnosed as AIS D compared to AIS C. This observation is supported by the correlation between motor evoked potentials amplitude, motor recovery and muscle coherence activity stimated in subjects with SCI identified during rehabilitation [15].

Subjects diagnosed within the AIS D classification group demonstrate different degrees of gait function, which is usually assessed by qualitative clinical tests in the rehabilitation setting [43,61]. It is of interest to note therefore that calculation of 15–30 Hz TA coherence activity estimated during isometric movement also correlates with the grade of gait function (WISCI II). Other studies have also shown the clinical utility of measurement of motor unit synchronization, corticospinal tract integrity and gait function after SCI [13,15], particularly as TA coherence activity within this frequency band is strongly reduced or absent after SCI [22].

### TA coherence estimation and SCI spasticity

The pathophysiology of spasticity following SCI is complex and most probably involves parallel changes in pyramidal, extrapyramidal and spinal motor control mechanisms [62-64], some of which are better evaluated during residual voluntary motor activity function [6]. Many symptoms of motor dysfunction have been associated with subjects with spasticity, including muscle hypertonia, spinal hyperreflexia, coactivation, spasms and clonus [6,8,63]. Although no difference was observed for TA coherence activity in subjects with or without spasticity during isometric dorsiflexion, correlational analysis revealed inverse relationships between motor unit synchronisation and several symptoms of spasticity within the low (10-16 Hz) and high (40-60 Hz) frequency bands. Specifically passive resistive torque in subjects with hypertonia inversely correlated with 10-16 Hz TA coherence activity, in contrast to the modified Ashworth or SCATS scores which negatively correlated with high frequency (40–60 Hz) motor unit synchronisation. Interestingly another study also associated low-frequency coherence activity with neuronal activity within spinal pathways [25,26], which would explain the relationship between TA coherence within this bandwidth and the tonic stretch reflex activity. Of passing interest here also is that clonus activity [65] measured in patients with spasticity was associated positively with 10-16 Hz TA coherence activity supporting the hypothesis that clonus and passive tonic stretch reflex activity measured in subjects with spasticity are predominantly mediated via different spinal mechanisms [66]

In contrast the association between high frequency 40-60 Hz TA coherence activity and the clinical measures of spasticity (modified Ashworth scale and SCATS) most probably reflects central neuronal activity unrelated to the 15-30 Hz activity that approximates pyramidal activity (see above). Indeed evidence suggests that lower limb hypertonia, as assessed with the modified Ashworth scale, may also be mediated by hyperexcitability of extrapyramidal neuronal mechanisms organized at the brainstem level [67].

### TA coherence activity as an approximation of adaptive and maladaptive neuroplasticity after SCI

The presence of both muscle hypertonia and evoked spasm activity in subjects with lower 40-60 Hz coherence following SCI also suggests that non-pyramidal adaptive mechanisms could mediate residual motor recovery, which would be masked by the development of spasticity in our cohort. Closer neurophysiological analysis of the activity within these pathways after SCI should be made to provide more detailed evidence regarding both maladaptive neuroplasticity related to spasticity [68] and adaptive neuroplasticity related to recovery of residual motor function [69,70].

It is interesting to observe the correlation between the time from SCI with 15–30 Hz and 24-40 Hz TA motor unit synchronisation estimated during fast isokinetic movement which suggests that a closer examination of velocity-dependent dorsiflexor muscle coherence activity would provide prognostic information regarding the potential for residual motor function recovery. Indeed coherence activity calculated within these mid-range frequency bands during maximal isometric contraction also correlate with gait function after SCI in our study. Whether recovery of bait function depends on the development of velocity-dependent activity of residual pyramidal and extrapyramidal motor control systems after incomplete SCI will need to be addressed with more sophisticated neurophysiological techniques.

### Clinical application and limitations of intramuscular TA coherence estimation

Measurement of TA motor unit synchronisation as an approximation for descending motor control activity in subjects with incomplete SCI within the neurorehabilitation setting is an obvious clinical goal. In addition the possibility that TA coherence activity could reflect residual motor control recovery with training or deterioration following the development of spasticity needs to be addressed in a closely controlled subacute longitudinal study of SCI. Further studies should more closely analyse the functional significance of a more detailed coherence spectra in the future with longer EMG signals which will lead to a more precise identification of the frequency bands. In addition analysis of EMG coherence in those subjects with SCI with low levels of muscle contraction should be preprocessed using rectification [41].

## Conclusion

Intramuscular TA coherence estimation calculated between 15-30 Hz or 40-60 Hz during isometric activation at 100% of MVT or during fast 120°/s isokinetic activation may provide important diagnostic information regarding the state of voluntary motor control mechanisms following incomplete SCI. In patients without spasticity these coherence bands may reflect recovery of residual motor control. In contrast the clinical diagnosis of muscle hypertonia and evoked involuntary muscle spasms negatively affect 40-60 Hz TA coherence estimation. Systematic analysis of TA motor unit synchronization during specific motor tasks within specific bandwidths provides a basis for the development of a quantitative diagnostic method that approximate adaptive and maladaptive lower limb residual motor control mechanisms and neuroplasticity during subacute SCI. Further studies in a larger cohort of subjects with incomplete SCI, using longer rectified EMG signals will be required to test this hypothesis, including corroborative data obtained from neurophysiological and functional longitudinal studies.

## Abbreviations

EMG: Electromyographic activity; SCI: Spinal cord injury; TA: Tibialis anterior; AIS: American spinal injury association impairment scale; MVT: Maximal voluntary torque; SCATS: Spinal cord assessment tool for spastic reflexes scale; MUAP: Motor unit action potential.

## Competing interests

The authors declare that they have no competing interests.

## Authors’ contributions

EBE participated in collecting the subject data, conducting data and statistical analysis, and helped to draft the manuscript. MA developed the software required to analyse EMG activity and muscle coherence analyses. CS helped collect the subject data and to draft the manuscript. DT and JLP helped to draft the manuscript and supervise the research. JT and JG-S conceived the study, interpreted the data, drafted the manuscript and critically revised it. All authors read and approved the final manuscript.
